# Phthalate Exposure During the Prenatal and Lactational Period Increases the Susceptibility to Rheumatoid Arthritis in Mice

**DOI:** 10.3389/fimmu.2020.00550

**Published:** 2020-04-03

**Authors:** Elena Elter, Marita Wagner, Lisa Buchenauer, Mario Bauer, Tobias Polte

**Affiliations:** ^1^Department of Environmental Immunology, UFZ-Helmholtz Centre for Environmental Research Leipzig-Halle, Leipzig, Germany; ^2^Department of Dermatology, Venerology and Allergology, Leipzig University Medical Center, Leipzig, Germany

**Keywords:** perinatal exposure, rheumatoid arthritis, phthalates, early programming, autoimmune disease

## Abstract

The prenatal and early postnatal period is highly sensitive to environmental exposures that may interfere with the developmental programming of the immune system leading to an altered disease risk in later life. To clarify the role of early influences in activation or exacerbation of autoimmune diseases like rheumatoid arthritis (RA) we investigated the effect of maternal exposure during the prenatal and lactational period of DBA/1 mice to the plasticizer benzyl butyl phthalate (BBP) on the development of RA in the offspring. Using a mild collagen-induced arthritis (CIA) model, maternal BBP-exposure increased both the prevalence and the severity of RA in the progeny compared to un-exposed dams. Additionally, maternal BBP exposure led to elevated serum IgG_1_ and IgG_2a_ level in the offspring and increased the IFN-γ and IL-17 release from collagen-re-stimulated spleen cells. Transcriptome analysis of splenocytes isolated from 3-week-old pups before RA-induction revealed considerable changes in gene expression in the offspring from BBP-exposed dams. Among them were *regulator of G-protein signaling 1 (rgs1), interleukin-7 receptor (il-7r)* and *CXC chemokine 4 (cxcr4)*, all genes that have previously been described as associated with RA pathology. In summary, our results demonstrate that perinatal exposure to BBP increases the susceptibility of the offspring to RA, probably via a phthalate-induced disturbed regulation of RA-relevant genes or signaling pathways.

## Introduction

Environmental influences have been shown to play a crucial role in the development of various diseases like allergic asthma, cancer, or metabolic diseases (1, 2). External factors such as chemicals, pathogens or stress may act as drivers of the individual's risk to develop disease and as triggers of underlying genetic predispositions (3). Recent findings indicate that particularly the prenatal and early postnatal period appear critical to environmental exposures, probably interfering with the developmental programming of the immune system or the physiological endocrine and metabolic signaling and thereby altering the disease risk in later life (3). Although the etiology of autoimmune diseases like rheumatoid arthritis (RA) is still unclear, genetical, immunological, hormonal but also environmental factors are considered to be important triggers (1). It has been shown that e.g., infections, vaccines, drugs, or stress may affect the immune system and thus play a role in the development of autoimmune diseases. Also occupational and other chemical exposures such as smoking, alcohol consumption, or diet are discussed as triggers for autoimmunity (4). However, the role of environmental exposures to hormonally active chemicals like endocrine disruptors in activation or exacerbation of RA has not yet been clarified and only few data exist demonstrating such chemicals as triggers for autoimmune diseases (5). In the present study we focused on phthalates, namely n-benzyl butyl phthalate, a representative of a group of chemicals commonly used as plasticizers in large quantities worldwide. This chemical is present in a wide range of consumer products like food packaging and plastics (6, 7). Humans are exposed to phthalates mainly through ingestion or inhalation throughout their entire life starting already *in utero* (8, 9). Phthalates have been found to affect the reproductive system (10) and to be associated with obesity and an increased diabetes risk (11). Furthermore, there is also evidence that maternal phthalate exposure may modulate the immune system inducing allergy-promoting effects in children (12, 13). Interestingly, there is almost no information about the impact of phthalates on the development of RA (14). One reason for the limited number of epidemiological studies addressing the role of environmental or chemical exposures in RA development might be the relatively low prevalence and the high age of first manifestation compared to e.g., allergic diseases, where the influence of the environment is very well-documented (2). However, it seems very likely that exposures to environmental factors including chemicals may induce comparable effects regarding the initiation and maintenance of autoimmune diseases like RA.

## Materials and Methods

### Mice

Female and male DBA/1 mice (6–8 weeks of age) were obtained from the Elevage Janvier Laboratory (Le Genest St Isle, France). Mice were bred and maintained in the animal facility at the University of Leipzig (Germany) under conventional conditions with 23°C room temperature, 60% humidity, and 12 h day/night rhythm. Control and BBP-exposed dams and pups were housed in polyphenylsulfone (PPS) cages as described previously (13). All mice received phytoestrogen-free diet (C1000 from Altromin, Lage, Germany) and water *ad libitum* from custom-built glass bottles to avoid contamination with BBP. All animal experiments were performed according to institutional and state guidelines. The Committee on Animal Welfare of Saxony approved animal protocols used in this study (TVV39/11; 11/18).

### Exposure to BBP and Induction of Mild Collagen-Induced Arthritis

**Figure 1:**
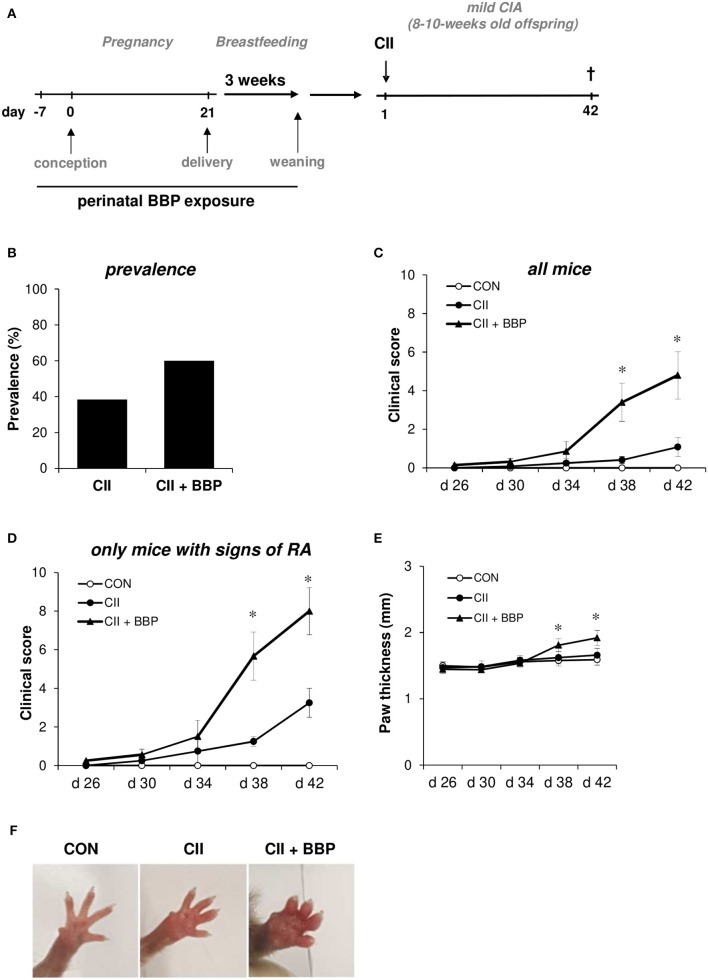
Perinatal exposure to BBP increases prevalence and the clinical of RA in the offspring. Dams were perinatally exposed to BBP and a mild collagen-induced Arthritis was induced in the offspring **(A)**. Effect of perinatal BBP exposure on RA prevalence **(B)**, on clinical severity scored on a scale of 1-4 for each paw of all mice **(C)** or only mice with signs of RA **(D)**, by measurement of paw thickness **(E)** and visualized by example pictures of affected hind limbs **(F)**. Data are expressed as mean ± SEM, *n* ≥ 13 animals per group; *p < 0.05, unpaired *t*-test.

To investigate the impact of an exposure during the prenatal and weaning period on the development of RA in the offspring, we exposed pregnant mice to BBP (3 μg/ml) exclusively via drinking water starting 1 week before mating until weaning when pups were 3 weeks old (perinatal exposure, Figure 1A). Control dams received normal drinking water. Just before delivery pregnant mice were separated into single cages.

Mild collagen-induced arthritis (CIA) was established in 8–10-week-old offspring from BBP-exposed or un-exposed dams (Figure 1A). Briefly, type II bovine collagen (bCII, EPC, Owensville, MO), solubilized in 0.05 N of acetic acid in sterile water at a concentration of 1 mg/mL was emulsified with an equal volume of complete Freund's adjuvant. Mice were immunized with 50 μl emulsion at the base of the tails on both sides (100 μl of emulsion total containing 50 μg bCII) on day 0. Control mice received the adjuvant without collagen. All mice were sacrificed on day 42. The development of arthritis was assessed three times weekly by two blinded observers. The clinical severity of arthritis in each paw was quantified according to a graded scale from 0 to 4, as follows: 0, no swelling; 1, swelling in one digit or mild oedema; 2, moderate swelling affecting several digits; 3, severe swelling affecting most digits; and 4, the most severe swelling and/or ankylosis. A total arthritis score per mouse was determined by summarizing the scores of all four extremities. Furthermore, paw swelling was assessed by measuring the thickness of the affected hind paws with 0–10 mm calipers.

### Measurement of IgG Antibodies and Cytokines

Serum anti-CII IgG_1_ and IgG_2a_ antibody levels were measured by sandwich ELISA on day 42 after immunization comparable to ELISA protocol described previously (15). To evaluate cytokine release splenocytes were re-stimulated with CII for 3 days. Cytokines were measured in supernatants using DuoSet® ELISA kits (R&D Systems, Minneapolis, USA) according to the manufacturer's instructions.

### RNA Extraction and Microarray Analysis

Total RNA was extracted from splenocytes from 3-week-old offspring by using RNeasy Mini Kit (Qiagen, Hilden, Germany) according to manufacturer's instructions. The mRNA transcription profiles of pooled samples (Supplementary Table 1) were determined by Clariom™ D assay for mouse (ThermoFisher Scientific, Schwerte, Germany). Microarray analysis was carried out by the Core Unit DNA-Technologies at the Medical Faculty of Leipzig University. Affymetrix GeneChip data representing ~214.000 transcripts were extracted from fluorescence intensities and were scaled in order to normalize data for interarray comparison using MAS 5.0 software according to the manufacturer's instructions (Affymetrix) (16).

### Bioinformatics Analysis

Annotation clustering was performed with DAVID bioinformatics resource 6.8 (17). For additional pathway analysis the KEGG Mapper was used (18). Principal component analysis (PCA) and heatmaps were created using SINGuLAR Analysis Tool (Fluidigm Corporation, CA).

### RT-PCR

For semi-quantitative PCR (qPCR), total RNA was prepared by using RNeasy Mini Kit (Qiagen, Hilden, Germany) according to manufacturer's instructions. The cDNA synthesis was carried out with 200 ng of RNA by using 5U RevertAid™ H Minus Reverse Transcriptase (Fisher Scientific, Schwerte, Germany), 20U Recombinant RNasin® Ribonuclease Inhibitor (Promega, Mannheim, Germany), 0.5 μl 28 μmol polyd(T)_12−18_ Primer (Carl Roth, Karlsruhe, Germany), 0.1 μg Random Hexamer Primer (Fisher Scientific), 0.2 μg dNTP-Set 1 (Carl Roth). Intron-spanning primers were designed using web-based Primer3 (19). The cycling program consisted of 95°C for 5 min, followed by 40 cycles of 95°C for 15 s and 60°C for 30 s and 72°C for 30 s on a LightCycler 480 (Roche Applied Science, Mannheim, Germany). The PCR mixture contained 1.25 mM MgCl_2_, 200 μM each dNTP, 0.24 μM of each primer (Supplementary Table 2), 1X Evagreen Dye (Biotrend, Köln, Germany) and 0.5 U of BIOTAQ DNA polymerase (Bioline, Luckenwalde, Germany). Gene expression values were determined by using the 2-ΔΔCT method (20) with *actb* and *rplp0* as reference genes and normalized to the lowest calculated value for each gene.

### Statistics

In mouse experiments Mann-Whitney and Wilcoxon signed-rank test were used to determine statistical differences between groups (GraphPad Prism 7.02, GraphPad Software, Inc.). Data were expressed as mean ± SEM and *P* values of < 0.05 were considered significant. Statistical significance of parametric distributed values was calculated with Student's *t*-test. Boxes in figures indicate the 25 and 75% percentile, whiskers the non-outlier range. Both sensitivity (proportion of positives that are correctly identified as such) and specificity (proportion of negatives that are correctly identified as such) as statistical measures of a binary classification test were calculated based on a cut-off or on clustering analysis. All *p* < 0.05 were considered to be significant. All statistical calculations were performed with Statistica for Windows version 10 (StatSoft Inc. Europe).

## Results

### Perinatal Exposure to BBP Increased the Prevalence and Severity of RA in the Offspring

To investigate the impact of maternal BBP exposure on the development of RA, dams were exposed to BBP during pregnancy and breastfeeding (perinatal). DBA/1/c mice received 3 μg/ml BBP via drinking water, a concentration shown to be relevant for the human exposure situation (13). In the offspring a mild RA was induced without further exposure to BBP (Figure 1A). Thirty eight percent of grown-up mice from un-exposed dams developed symptoms of RA, while the prevalence in the offspring from BBP-exposed dams was increased to 60% (Figure 1B). The increased prevalence seems to be primarily apparent in the female offspring (unexposed CII-treated mice: 33%, CII-treated mice from BBP-exposed dams: 66%), while the male offspring showed only a slight difference (CII: 50%, CII + BBP: 55%). However, the number of male pups in the un-exposed offspring was very limited (*n* = 4), which is why this finding has to be interpreted with caution. Consequently, we included the data of both sexes for all following RA parameters.

Perinatal BBP exposure significantly increased the clinical severity in the offspring quantified by the total arthritis score of all animals (Figure 1C). Considering only mice with signs of arthritis on the paws, the severity was still clearly enhanced in the offspring from BBP-exposed dams compared to mice from un-exposed dams (Figure 1D).

**Figure 2:**
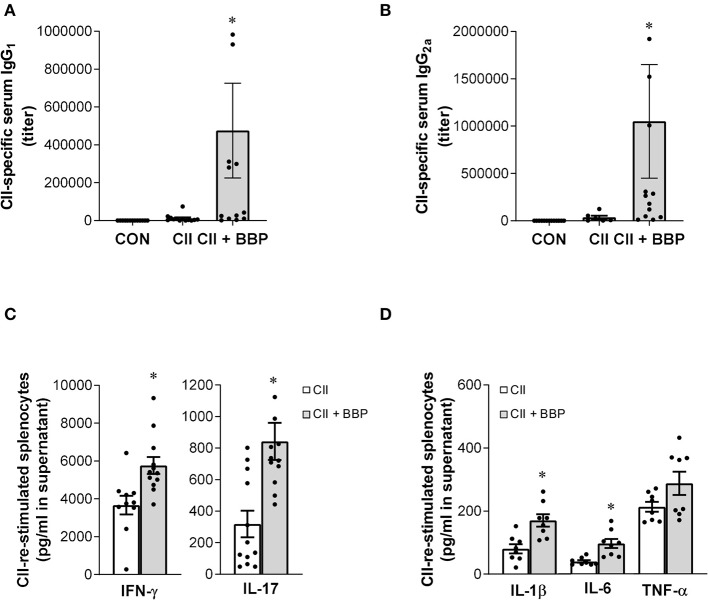
Perinatal exposure to BBP increases IgG antibody level, induces IFN-γ and IL-17 production and the release of pro-inflammatory cytokines in collagen-treated offspring. Effect of an exposure of CII-immunized DBA/1 mice to BBP on IgG_1_ **(A)** and IgG_2a_ antibody levels **(B)**, and on IFN-γ and IL-17 production **(C)** and the release of IL-1β, IL-6, and TNF-α in CII-re-stimulated splenocytes **(D)**. Data are expressed as mean ± SEM, *n* ≥ 13 **(A,B)** and *n* ≥ 8 **(C,D)** animals per group; *P < 0.5, unpaired *t*-test.

In addition, the paw thickness was elevated in the offspring from BBP-exposed dams (all mice, Figure 1E), also illustrated by photographic pictures of paws of the different groups on day 42 (Figure 1F). Furthermore, collagen-specific IgG_1_ and IgG_2a_ levels in serum were significantly enhanced in the offspring from BBP-exposed dams (Figures 2A,B).

The evaluation of cytokine release demonstrated that the increased arthritis severity in the offspring from BBP-exposed dams was accompanied by an elevated production of IL-17 and IFN-γ (Figure 2C) as well as the inflammatory cytokines IL-1β and IL-6 in the supernatant of CII-restimulated splenocytes, while the effect on TNF-α production was not significant (Figure 2D).

### Perinatal Exposure to BBP Induced an Altered Expression of RA-Related Genes in the Offspring

To investigate the effect of maternal BBP exposure on gene expression in the offspring we first performed a transcriptome analysis using Clariom™ D microarray with pooled samples, stratified by sex, and verified expression level for top-ranked differentially expressed genes by RT-PCR at individual level. Gene expression was performed on isolated splenocytes of 3-weeks old pubs before induction of the RA phenotype by immunization with collagen. Considering a 2-fold expression difference, in total, expression of 46 genes was sex-independently altered by BBP exposure (Table 1) with about three times as much under- (n, 35) in contrast to overexpressed (n, 10) genes. The highest degree did not exceed a 4-fold expression difference. Apart from three under-expressed genes of helicase (*mcm*2,-5,-6), genes were not enriched for their molecular function or role in cellular pathways (Supplementary Tables 3, 4). However, there was a striking accumulation of references (for 7 out of 10 BBP-induced overexpressed genes) showing an association of gene's overexpression with arthritis (Table 2).

**Table 1:** List of > 2-fold differentially expressed genes from Clariom™ microarray.

**Gene symbol**	**Gene description**		**Female**			**Male**		**ALL**
		**Control** **(*n*, 12)**	**BBP** **(*n*, 6)**	**BBP/CON**	**Control** **(*n*, 4)**	**BBP** **(*n*, 4)**	**BBP/CON**	**BBP/CON**
*Prg4*	Proteoglycan 4 (megakaryocyte stimulating factor)	128	717	5.6	172	394	2.3	4.0
*Tnfaip3*	Tumor necrosis factor alpha-induced protein 3	2,427	7,417	3.1	2,555	8,162	3.2	3.2
*Il7r*	Interleukin 7 receptor	7,401	22,170	3.0	6,748	13,808	2.0	2.5
*Rgs1*	Regulator of G-protein signaling 1	2,241	6,365	2.8	1,602	4,810	3.0	2.9
*Ddit4*	DNA-damage-inducible transcript 4	3,117	8,200	2.6	1,921	4,235	2.2	2.4
*Egr1*	Early growth response 1	553	1,442	2.6	656	1,550	2.4	2.5
*Sik1*	Salt inducible kinase 1	1,831	4,382	2.4	1,610	4,507	2.8	2.6
*Slxl1*	Slx-like 1	75	162	2.2	60	121	2.0	2.1
*Cxcr4*	Chemokine (C-X-C motif) receptor 4	31,351	63,624	2.0	31,380	65,758	2.1	2.1
*Rgs16*	Regulator of G-protein signaling 16	180	352	2.0	213	485	2.3	2.2
*Slc38a5*	Solute carrier family 38 member 5	285	142	−2.0	370	118	−3.1	−2.6
*Gfi1b*	Growth factor independent 1B	359	177	−2.0	490	194	−2.5	−2.3
*Chchd1*	Coiled-coil-helix-coiled-coil-helix domain containing 1	180	89	−2.0	199	92	−2.2	−2.1
*Tubb4b*	Tubulin beta 4B class IVB	321	157	−2.0	360	172	−2.1	−2.1
*Cox5b*	Cytochrome c oxidase subunit Vb	954	482	−2.0	1,172	581	−2.0	−2.0
*Pkhd1l1*	Polycystic kidney and hepatic disease 1-like 1	260	122	−2.1	549	167	−3.3	−2.7
*Car2*	Carbonic anhydrase 2	75,967	36,138	−2.1	119,780	39,466	−3.0	−2.6
*Ndufa4*	NADH dehydrogenase (ubiquinone) 1 alpha subcomplex 4	2,097	1,001	−2.1	3,390	1203	−2.8	−2.5
*Mcm2*	Minichromosome maintenance deficient 2 mitotin	1,668	780	−2.1	2,136	860	−2.5	−2.3
*Gzma*	Granzyme A	2,176	1,041	−2.1	2,166	920	−2.4	−2.3
*Mcm6*	Minichromosome maintenance deficient 6	2,641	1,260	−2.1	3,278	1,556	−2.1	−2.1
*Nme1*	NME/NM23 nucleoside diphosphate kinase 1	196	91	−2.1	211	99	−2.1	−2.1
*Slirp*	SRA stem-loop interacting RNA binding protein	474	231	−2.1	602	287	−2.1	−2.1
*Ccr3*	Chemokine (C-C motif) receptor 3	1,868	883	−2.1	1,481	734	−2.0	−2.1
*Aqp1*	Aquaporin 1	4,664	2,107	−2.2	8,320	2,278	−3.7	−3.0
*Fads3*	Fatty acid desaturase 3	368	171	−2.2	571	165	−3.5	−2.9
*Asns*	Asparagine synthetase	515	231	−2.2	584	212	−2.8	−2.5
*2010107E04Rik*	RIKEN cDNA 2010107E04 gene	807	369	−2.2	1,069	443	−2.4	−2.3
*Hebp1*	Heme binding protein 1	496	212	−2.3	1,042	325	−3.2	−2.8
*Mrpl42*	Mitochondrial ribosomal protein L42	3,593	1,583	−2.3	4,678	1,760	−2.7	−2.5
*Rrm2*	Ribonucleotide reductase M2	2,283	1,013	−2.3	2,960	1,153	−2.6	−2.5
*Ifi27l2a*	Interferon alpha-inducible protein 27 like 2A	322	143	−2.3	445	180	−2.5	−2.4
*Mcm5*	Minichromosome maintenance deficient 5	3,013	1,312	−2.3	3,230	1,542	−2.1	−2.2
*Mt2*	Metallothionein 2	5,404	2,312	−2.3	4,389	2,105	−2.1	−2.2
*Cdc6*	Cell division cycle 6	387	162	−2.4	474	192	−2.5	−2.5
*Cxcl9*	Chemokine (C-X-C motif) ligand 9	287	118	−2.4	266	126	−2.1	−2.3
*Cpox*	Coproporphyrinogen oxidase	1,339	544	−2.5	1,879	930	−2.0	−2.3
*S100a6*	S100 calcium binding protein A6 (calcyclin)	196	77	−2.5	248	122	−2.0	−2.3
*Nt5dc2*	5-nucleotidase domain containing 2	570	218	−2.6	581	225	−2.6	−2.6
*Car1*	Carbonic anhydrase 1	58,091	22,544	−2.6	73,080	29,328	−2.5	−2.6
*Ccl6*	Chemokine (C-C motif) ligand 6	1,039	402	−2.6	954	457	−2.1	−2.4
*Cldn13*	Claudin 13	393	141	−2.8	824	210	−3.9	−3.4
*Klf1*	Kruppel-like factor 1 (erythroid)	174	63	−2.8	213	74	−2.9	−2.9
*Alox15*	Arachidonate 15-lipoxygenase	939	299	−3.1	376	143	−2.6	−2.9
*Pklr*	Pyruvate kinase liver and red blood cell	675	196	−3.5	546	190	−2.9	−3.2

*Compared were gender-dependent pools of 3-weeks-old offspring from perinatal BBP-exposed dams with controls. Gender-independent up- and downregulated genes were dissimilar distributed with less up- (22%) than downregulated (78%) genes*.

**Table 2:** Verification of differential gene expression in offspring from microarray (Mouse Clariom™ D assay) on pooled samples by RT-PCR at individual level.

**Mouse Clariom**™ **D assay**			**RT-PCR (n-control, 14; n-bbp, 10)**			
**Probe Set ID**	**Gene**	**RNA-Pool**	**Ensembl-ID**	**BBP vs. Control**		
		**x-fold**	**ENSMUSG000000**	***p*-value (ttest)**	**x-fold**	**Reference for “Arthritis”**
TC0100003029.mm.1	*cxcr4*	2.1	45382	**0.002**	1.7	(21)
TC1000002224.mm.1	*ddit4*	2.4	20108	**0.009**	2.0	(22)^*^
TC1800000301.mm.1	*egr1*	2.5	38418	0.25	1.7	(23)
TC1500001197.mm.1	*il7r*	2.5	3882	**7.8E-05**	1.8	(24, 25)
TC0100003240.mm.1	*prg4*	3.9	6014	**0.020**	3.5	(26)
TC0100003207.mm.1	*rgs1*	2.9	24042	**6.3E-06**	3.2	(27)
TC0100001381.mm.1	*rgs16*	2.1	26475	0.23	1.4	
TC1700001827.mm.1	*sik1*	2.6	24042	**3.4E-06**	2.1	(28)
TC1000001813.mm.1	*tnfaip3*	3.1	19850	0.09	1.5	(29, 30)
TC1100003093.mm.1	*alox15*	−2.9	18924	0.11	1.5	(31)^*^
TC0300001667.mm.1	*car1*	−2.5	27556	0.94	−1.0	
TC1100003399.mm.1	*ccl6*	−2.3	18927	0.84	−1.0	
TC0500003382.mm.1	*cldn13*	−3.4	8843	0.12	−1.9	
TC1600000763.mm.1	*cpox*	−2.2	22742	0.25	−1.3	
TC0500002755.mm.1	*cxcl9*	−2.3	29417	0.19	−1.2	
TC0800000971.mm.1	*klf1*	−2.8	54191	0.055	−2.0	
TC1400000322.mm.1	*nt5dc2*	−2.6	71547	**0.007**	−1.4	
TC0300000747.mm.1	*pklr*	−3.2	41237	**5.4E-05**	−2.4	

Gene expression was measured on fourteen 3-week-old mice from control and ten mice from BBP-exposed dams. RT-PCR overall confirms direction of gene expression and partially reaches significant differences between groups. Remarkably, genes associated with arthritis were enriched.

* Association of downregulation with arthritis.

*Statistical significance is indicated in bold (p < 0.05)*.

**Figure 3:**
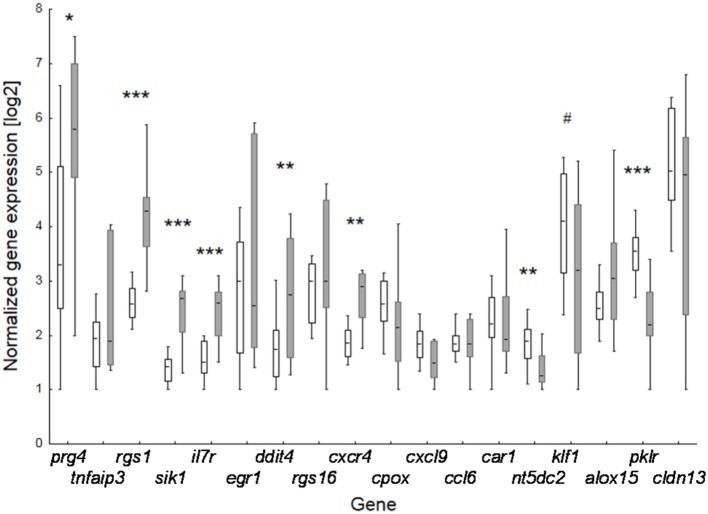
Effect of perinatal BBP exposure on gene expression in 3-weeks old offspring. Verification of data of the 18 top-ranked differentially expressed genes obtained by microarray analysis for pooled samples by RT-PCR at individual level. Boxes for controls (CON, white; *n* = 14) and BBP (gray; *n* =10) indicate mean and the 25 and 75% percentile, whiskers the non-outlier range. *P-value* (*t*-test): ^#^0.055; * <0.05; ** <0.01; *** <0.001.

**Figure 4:**
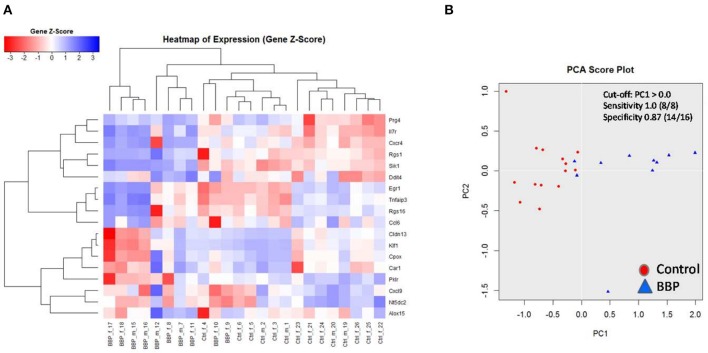
Clustering of 18 top-ranked differential gene expression in spleen of 3-weeks old offspring after perinatal BBP exposure in dams. Gene expression was performed by RT-PCR. Both heatmap **(A)** and principal component analysis (PCA) **(B)** highlight a strong stratification into control (CON) and BBP. Counts from 1 to 24 are identification numbers (ID) of offspring. F, female; m, male.

To verify microarray data for pooled samples, RT-PCR at individual level was performed for 18 top-ranked differentially expressed genes (9 up- and 9 down-regulated genes). For 6 up- and 3 (1 by trend) down-regulated genes the BBP-induced alteration in gene expression was confirmed (Table 2 and Figure 3). Both clustering analysis for all 18 analyzed genes by RT-PCR as well as PCA analysis showed a strong stratification of offspring by mother's perinatal BBP exposure with a sensitivity of 1.0 and specificity of 0.87 (Figures 4A,B).

## Discussion

In recent years phthalates have been associated with an increased prevalence of diseases like obesity and allergies or shown to affect the cardiovascular and reproductive system (10, 11, 32). In particular, exposure during pregnancy and early childhood seems to be a risk factor for enhanced disease susceptibility in children (3). Although several studies described immune-modulating effects of maternal phthalate exposure leading e.g., to an enhanced risk for atopic dermatitis (12) or allergic asthma (13) in early childhood, there are no information from epidemiological nor experimental studies about a possible role of phthalates on RA development. Here, we show that perinatal exposure to BBP increased both the RA prevalence and the clinical severity in the offspring. For this, we intentionally used a mild CIA model in DBA/1 mice with relatively low prevalence and only little signs of RA-related symptoms to underline the importance of the environmental factor for the disease outcome. As described earlier, the applied low-dose BBP were based on the tolerable daily intake of BBP in humans of 0.5 mg/kg body weight/day (33), whereby the urinary concentration of the BBP metabolite mono-n-butyl phthalate in the murine model corresponded with those observed in highly exposed mothers in our prospective mother-child cohort LINA (13). This approach establishes a scenario that is representative for the real exposure situation in humans.

While there are no data regarding phthalate exposure and RA development, this group of chemicals has been reported to affect other autoimmune diseases. In mice, exposure to dibutyl phthalate aggravated autoimmune thyroid disease and—comparable to our results—induced an increased release of the pro-inflammatory cytokines IL-1β, IL-6, and IL-17 (34, 35). Another study demonstrated that low-dose exposure to di-(2-ethylhexyl) phthalate (DEHP) increased the susceptibility to testicular autoimmunity (36). The effect was accompanied by an elevated number of IFN-γ-positive cells. Moreover, DEHP has been shown to induce the production of the RA-relevant cytokines IL-1β, IL-6, and TNF-α in macrophages (23, 37). Therefore, despite missing data in regard to phthalate-induced effects on initiation or maintenance of RA, there are indications that phthalate exposure may affect Th1 (IFN-γ) or Th17 (IL-17) cell activity and the production of disease-relevant pro-inflammatory cytokines that can also promote the development of RA.

As a first step to understand why DBA/1 mice from phthalate-exposed dams show a higher susceptibility to RA development, we investigated possible alterations in gene expression in splenocytes of 3-week-old offspring from perinatally BBP-exposed dams before induction of CIA. We initially analyzed pooled individual samples, stratified by gender and exposure, by microarray and subsequently verified top-ranked differentially expressed genes by RT-PCR at individual level. We found genes that convincingly indicate the perinatal BBP-exposure of offspring's dams. Although the cellular origin of altered gene expression in spleen was not resolved in detail in this study, we suppose a prominent BBP-induced effect on immunocompetent cells in this tissue. The pattern of the strongest differentially-expressed genes did not enrich specific genes neither of special immunocompetent cells of the spleen nor general pathways, but interestingly, these genes were frequently found to be associated with arthritis. Among them were genes like *regulator of G-protein signaling 1 (rgs1), interleukin-7 receptor (il-7r)*, and *CXC chemokine 4 (cxcr4.)* As expected, most of the identified arthritis-associated genes in the immune organ spleen have been found not exclusively in arthritic joints but also in immunocompetent cells of peripheral blood. Regarding sex-dependent changes in gene expression by BBP, there were additional sex-specific changes. As the majority of such changes relied on initial differences in controls or on Y-chromosomal linkage in male, they were not further prioritized in this study.

The physiological role of *rgs1* as the most discriminating gene to distinguish BBP-exposed dams in offspring (sensitivity 1.0, specificity 0.94) within splenocytes is not clear yet. It has been found in different immunocompetent cells. Rgs1 knock-out mice have an impaired B-cell migration in response to chemokines (38). Together with *rgs16* it was identified to be more abundant in activated regulatory CD4^+^ T-cells than in non-activated T-cells (39). Apart from splenocytes, it was described as the most useful biomarker distinguishing especially undifferentiated spondyloarthritis and, to a lesser extent, ankylosing spondylitis in human peripheral blood mononuclear cells (PBMC). It was suggested to be present in monocytes, in which *rgs1* was inducible by arthritis-causing cytokines, like TNF-α and IL-17 (27). IL-7 receptor (gene *il7r*), as a pro-inflammatory-acting receptor, is associated with arthritis for immunocompetent cells both of adaptive and innate immune system. It was shown, that IL-7 stimulated IL-7R+ mature B cells act pro-inflammatory (increased clinical score, increased anticollagen type II antibodies) after cell transfer in collagen type II-induced arthritis in DBA/1 mice (24). In humans, IL7R-expressing macrophages where found to be elevated in both RA synovial fluid as well as peripheral blood (25). C-X-C chemokine receptor type 4 (gene *cxcr4*) on lymphocytes is involved in inflammatory processes. In human PBMC, RA patients displayed a higher frequency of CXCR4^+^ memory T cells which were additionally associated with disease severity (21).

The results from the transcriptome analysis were surprising and represent cause for concern regarding the transgenerational impact of maternal lifestyle (BBP-consumption) on gene expression of the progeny. However, it must be noted that DBA/1 mice are particularly susceptible to CIA because of their specific genotype (40). This also illustrates that an interaction between a susceptible genotype and environmental factors can act as trigger for disease initiation, which has also been discussed for RA (4). The altered gene expression might be mediated by early epigenetic changes induced by BBP exposure during pregnancy and the breastfeeding period and should be investigated in further studies. BBP-induced epigenetic modifications have already been described in relation to its asthma-promoting effects, further emphasizing that the developing immune system is especially susceptible to perturbations by external factors (13).

In summary, our study results show that perinatal exposure to BBP increases the susceptibility of the offspring to the development of RA. The effect might be mediated via a phthalate-induced disturbed regulation of RA-relevant genes. Our findings indicate that early exposure to plasticizers, such as phthalates, represent not only a risk factor for obesity or allergies but also for the development of autoimmune diseases like RA.

## Data Availability Statement

Raw sequencing data with appropriate experimental information is available in the NCBI Gene Expression Omnibus (GEO) repository under the accession number GSE146699. Other raw data supporting the conclusions of this article will be made available by the authors, without undue reservation, to any qualified researcher.

## Ethics Statement

The animal study was reviewed and approved by the Committee on Animal Welfare of Saxony/Leipzig, Germany.

## Author Contributions

EE, MW, and LB performed the mouse experiments. TP designed and conducted the study and experiments. MB analyzed the microarray data and conducted the PCR analysis. TP and MB wrote the paper with substantial inputs from LB.

### Conflict of Interest

The authors declare that the research was conducted in the absence of any commercial or financial relationships that could be construed as a potential conflict of interest.
